# Case-study: Patient with acquired epileptic aphasia in childhood

**DOI:** 10.1192/j.eurpsy.2024.945

**Published:** 2024-08-27

**Authors:** P. Terziivanova, A. Ushkova, K. Petkova, D. Borisova, A. Popova, P. Marinov

**Affiliations:** ^1^ Mental Health Centre “Prof. Nikola Shipkovenski” LTD; ^2^Sofia University “St Kliment Ohridski”, Medical Department of Psychiatry, Sofia, Bulgaria

## Abstract

**Introduction:**

Acquired epileptic aphasia or Landau-Kleffner syndrome (LKS) is a disorder with onset in the childhood between the ages of 2 and 8 years. The main defining psychopathological symptom of Landau-Kleffner syndrome is the acquired aphasia with epileptiform electroencephalographic abnormalities. The aphasia has both receptive and expressive features. The onset is usually subacute and the course is usually progressive with spontaneous improvements and exacerbations. The electroencephalographic abnormalities include pathological findings in the temporal and parieto-occipital brain regions.

**Objectives:**

An 11 year old girl with generalized tonic-clonic and partial seizures is referred to our child and adolescence outpatient service due to language impairment. Her first generalized seizure has been at the age of 11 months old, caused by high temperature. The presence of articulation difficulties has raised suspicion for intellectual disabilities. She has been diagnosed with Epilepsy, grand mal seizures and has had continuous treatment with sodium valproate since the age of 3 years.

**Methods:**

We used medical history, EEG-recordings, clinical observation and psychological assessment.

**Results:**

Patient`s language development has been normal till the age of 3 years old. She has started using single words properly at the age of 1 year and 6 months old. Her first simple sentences have appeared at the age of 2 years old. At the age of 3 years old after severe generalized tonic-clonic seizures she has stopped talking for a month. After this month she had started vocalizing and using simple words, but she had lost her ability to form sentences. She has had some mild difficulties in understanding verbal information and following instructions. Her speech has had bad articulation and deficits in the verbal fluency. Her gross and fine motor development, her social skills and problem-solving abilities have all been intact and age-appropriate. She has worked with speech therapist for 5 years and achieved partial recovery from the acquired aphasia. She continues to have problems with the articulation – the speech is still with mild dysarthria. We used WISC-IV to assess her IQ (IQ=108).

**Conclusions:**

The patient has already developed age-appropriate speech prior to the onset of the language impairment. Considered as secondary or acquired, the observed aphasia together with the medical data for her epileptic seizures allows us to diagnose the patient with Acquired epileptic aphasia or Landau-Kleffner syndrome. Later development will be presented and discussed.

**Disclosure of Interest:**

None Declared

